# Facile Cell-Friendly Hollow-Core Fiber Diffusion-Limited Photofabrication

**DOI:** 10.3389/fbioe.2021.783834

**Published:** 2021-12-03

**Authors:** Alexander G. Savelyev, Anastasia V. Sochilina, Roman A. Akasov, Anton V. Mironov, Alina Yu. Kapitannikova, Tatiana N. Borodina, Natalya V. Sholina, Kirill V. Khaydukov, Andrei V. Zvyagin, Alla N. Generalova, Evgeny V. Khaydukov

**Affiliations:** ^1^ Federal Scientific Research Centre “Crystallography and Photonics” Russian Academy of Sciences, Moscow, Russia; ^2^ Center of Biomedical Engineering, Institute of Molecular Medicine, Sechenov University, Moscow, Russia; ^3^ Shemyakin-Ovchinnikov Institute of Bioorganic Сhemistry RAS, Moscow, Russia; ^4^ MQ Photonics Centre, Faculty of Science and Engineering, Macquarie University, Sydney, NSW, Australia

**Keywords:** hyaluronic acid, flavin mononucleotide, hollow-core fiber, cell-laden hydrogel, photopolymerization, photofabrication, radical diffusion, vessel engineering

## Abstract

Bioprinting emerges as a powerful flexible approach for tissue engineering with prospective capability to produce tissue on demand, including biomimetic hollow-core fiber structures. In spite of significance for tissue engineering, hollow-core structures proved difficult to fabricate, with the existing methods limited to multistage, time-consuming, and cumbersome procedures. Here, we report a versatile cell-friendly photopolymerization approach that enables single-step prototyping of hollow-core as well as solid-core hydrogel fibers initially loaded with living cells. This approach was implemented by extruding cell-laden hyaluronic acid glycidyl methacrylate hydrogel directly into aqueous solution containing free radicals generated by continuous blue light photoexcitation of the flavin mononucleotide/triethanolamine photoinitiator. Diffusion of free radicals from the solution to the extruded structure initiated cross-linking of the hydrogel, progressing from the structure surface inwards. Thus, the cross-linked wall is formed and its thickness is limited by penetration of free radicals in the hydrogel volume. After developing in water, the hollow-core fiber is formed with centimeter range of lengths. Amazingly, HaCaT cells embedded in the hydrogel successfully go through the fabrication procedure. The broad size ranges have been demonstrated: from solid core to 6% wall thickness of the outer diameter, which was variable from sub-millimeter to 6 mm, and Young’s modulus ∼1.6 ± 0.4 MPa. This new proof-of-concept fibers photofabrication approach opens lucrative opportunities for facile three-dimensional fabrication of hollow-core biostructures with controllable geometry.

## Graphical Abstract

**Figure:**
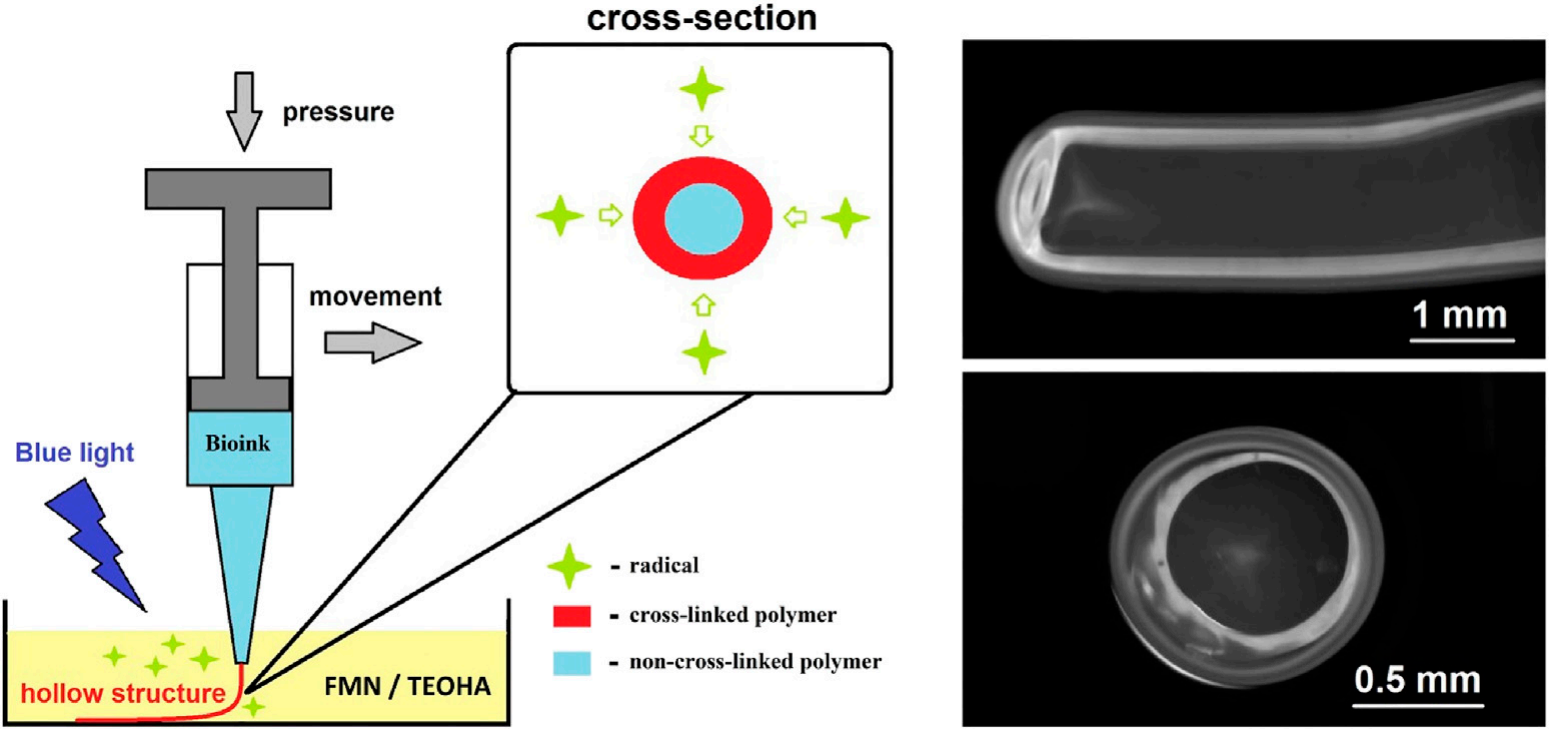


## Introduction

Three-dimensional (3D) printing is widespread additive manufacturing technology for fabricating spatially precise structures highly demanded in regenerative medicine. Synthetic micro- or macrostructures seeded with viable cells can be used either for implantation, drug screening, or toxicity testing (Blaeser et al., 2017; Marrella et al., 2019). To date, 3D-printing systems including inkjet printing (Pereira et al., 2017; Negro et al., 2018), extrusion printing (Spencer et al., 2019), and stereolithography (Jang et al., 2016) have been demonstrated for prototyping of living tissues and even whole organs.

Hydrogel hollow-core fibers represent a broad class of ubiquitous biomimetic structures, such as biomimetic blood vessels and bile ducts, and are highly demanded for many applications in tissue engineering. At the same time, the process of microfabrication of such structures remains challenging as exemplified by the reported fabrication of hollow-core fibers using complicated multi-stage alginate-based fluidic technologies (Distler et al., 2019; Wang et al., 2019; Nie et al., 2020). Facile single-step printing of implantable structures, containing living cells, as well as design and synthesis of cell-friendly inks are desirable and form a persistent trend in modern biofabrication (Rana et al., 2014). Since photocuring represents the most precise approach to form micro- and macrostructures, a careful selection of polymer and photoinitiator as the key components of the inks is required. To this aim, modified naturally derived polymers based on conjugates of gelatin (Suvarnapathaki et al., 2018), collagen (Gentile et al., 2017), chitosan (Sultankulov et al., 2019), and hyaluronic acid (HA) (Kufelt et al., 2014) are the best candidates to co-realize tunable mechanical and biocompatible properties. To meet stringent biomedical requirements, a photoinitiator must be water miscible, feature lack of dark cytotoxicity and moderate light toxicity, and have non-toxic photoproducts and preferable properties of photoactivation in visible or near-infrared spectral ranges.

Overcoming the combination of the above-mentioned limitations is a challenge for contemporary biomimetic hollow-core structures production. To address this problem, we developed conceptually new approach for cell-friendly hydrogel gelation *via* water-mediated diffusion of free radicals for which we coined a term “diffusion-limited photofabrication”. This approach is based on hydrogel gelation, while the width of cross-linked layer strictly depends on the penetration depth of free radicals caused by soaking and diffusion. The free radicals generated in photoinitiator water solution under light irradiation initially penetrate from the liquid phase to the extruded ink. This results in cross-linking (polymerization) of the hydrogel ink at the rim. Hyaluronic acid glycidyl methacrylate (HAGM)–based bioink and flavin mononucleotide/triethanolamine (FMN/TEOHA) combination as a source for generation of free radicals under 450 nm light irradiation were utilized for idea realization. After cross-linking all samples were subjected to developing in water. The hollow-core fibers were formed with centimeter range of lengths and retention of liquid flow within the structure was demonstrated. HaCaT cells embedded in the hydrogel successfully go through the fabrication procedure. Viability of embedded cells has been confirmed during 6 days. Varying the diameter of needles and time of cross-linking, we produced hydrogel fibers in a broad size range aiming to demonstrate unique opportunities for flexible design of biomimetic hollow-core structure initially embedded with living cells.

## Materials and Methods

### Materials

The following materials were purchased from Sigma-Aldrich (United States) and used without further purification: sodium hyaluronate (M_n_ = 100 kDa), glycidyl methacrylate (GMA), tetraethylammonium bromide (TEAB), poly(ethylene glycol) diacrylate (PEG-DA, M_n_ = 575), acetone, N,N-dimethylformamide (DMF), potassium permanganate (KMnO_4_), sodium tetraborate decahydrate (Na_2_B_4_O_7_×10H_2_O), concentrated hydrochloric acid (36%), glacial acetic acid, 4-(dimethylamino)benzaldehyde (DMAB), N-acetyl-D-glucosamine (NAG), and hyaluronidase from bovine testes. Flavin mononucleotide (FMN) was obtained from Pharmstandard (Russia). Triethanolamine (TEOHA) was purchased from Merck (United States). 2,2-Diphenyl-1-picrylhydrazyl (DPPH), 97% purity, was obtained from Hangzhou Dingyan Chem Co., Ltd (China). Phosphate buffered saline (PBS), pH 7.4, was prepared by dissolving biotechnology grade PBS tablet (VWR Life Science, Canada) in 100 ml of deionized water. Penicillin–streptomycin (5,000 U/ml and 5,000 µg/ml, respectively) was purchased from PanEco (Russia). Amphotericine B (5,000 µg/ml) was purchased from JSC “Sintez” (Russia).

### Bioink Synthesis

#### Modification of Hyaluronic Acid With Glycidyl Methacrylate

The approach is based on the protocol described by us earlier in Sochilina et al. (2019). Modification of hyaluronic acid with methacrylate containing reagents was chosen due to the reported mild cytotoxicity of the conjugated groups, simple one-step modification process, and large number of studies covering this type of modification (Collins and Birkinshaw, 2013; Poldervaart et al., 2017). HA in the form of sodium hyaluronate (1 g) was dissolved in 128 ml of deionized water under vigorous stirring. After dissolution, TEAB, as a phase-transfer catalyst, was added with the mass ratio HA:TEAB = 1:0.64. Then, 80 ml of DMF was added with consequent dissolution of GMA. Also, 650 μl of penicillin–streptomycin (5,000 U/ml and 5,000 µg/ml, PanEco) and 150 µl of amphotericin B (5,000 µg/ml, Russia) were used to prevent possible growth of microorganisms.

In order to achieve degrees of substitution (DS) of HAGM in the range from 11 to 69% different mass (g) to volume (ml) ratios of HA:GMA varying from 1:5 to 1:35 were used. Also, the variation of DS was accomplished by alteration of reaction time from 1 to 5 days. All reactions were carried out under continuous stirring at 40°C. In order to stop the reaction, hyaluronic acid, modified with glycidyl methacrylate (HAGM), was precipitated in a 7-fold excess of acetone. Then, HAGM was dialyzed against deionized water to purify from unreacted substances and was lyophilized using the FreeZone Freeze Dry System (Labconco, United States).

#### Evaluation of Vinyl Group Content in Hyaluronic Acid Glycidyl Methacrylate

The double bond concentration in HA after modification with GMA was determined by using the method described by us in Sochilina et al. (2019). In brief, HAGM was dissolved in deionized water at concentration 5 mg/ml and then added to the standard solution of titrant KMnO_4_ (1 ml, 0.25 mg/ml) until the color was changed from purple to brownish yellow. Complete reduction of permanganate ions was confirmed spectrophotometrically by absorption peaks disappearance in 420–600 nm range. DS of HA functional groups by GMA was evaluated as the ratio of vinyl group concentration to HAGM disaccharide unit concentration. All the absorption spectra were recorded at UV-Vis Spectrophotometer Evolution 201 (Thermo Fisher Scientific, United States).

The degree of substitution *DS* was found as we described before in Sochilina et al. (2019):

$$DS=\frac{C_{m}\left(GMA\right)}{C_{m}\left(disach\right)}\times 100\;\left(\text{\%}\right)\text{,}$$ (1)

where $C_{m}\left(GMA\right)$ is the molar concentration of GMA moieties (mM) required for complete reduction of standard solution of titrant, and $C_{m}\left(disach\right)$ is the general molar concentration of disaccharides (mM) containing in analyzed sample that fully reduced KMnO_4_.

#### Cell Culturing

Human keratinocytes HaCaT cells were obtained from Sechenov University cell lines collection (ATCC originally). The cells were cultured in Dulbecco’s Modified Eagle Medium (Gibco, United States) supplemented with 10% fetal bovine serum (HyClone, United States), 2 μМ L-glutamine, 100 μg/ml streptomycin, and 100  U/ml penicillin (Gibco, United States) at 37°C in a 5% CO_2_ humidified atmosphere. Once the cells reached ∼80–90% confluence, they were trypsinized; the subcultivation ratio was 1:6 to 1:8.

#### Bioink Preparation

As-synthesized HAGM and PEG-DA were dissolved in PBS under sonication to obtain a homogeneous solution. The mass fractions of HAGM and PEG-DA in solution were 25 and 6.25 wt% in relation to composition mass, correspondingly. Previously, human keratinocytes HaCaT were harvested with trypsin (0.05%) and centrifuged (300 g, 5 min). The cell pellet was resuspended in 200 µl of full DMEM. Polymer solutions were mixed with growing medium containing cells with the mass ratio 0.8:0.2 and carefully stirred. Finally, 1 g of bioink contained one million of HaCaT cells. As a result, the concentrations of HAGM and PEG-DA were reduced to 20 and 5 wt% in relation to composition, correspondingly. As we described earlier (Savelyev et al., 2017), this concentration is sufficient to meet the requirement of entire hydrogel volume formation indicating mechanical properties after cross-linking.

### Rheological Study

The rheological testing routine was performed using viscometer Brookfield DV2T (Ametek Brookfield, United States) equipped with low volume cone-plate measuring system CP-51. Prior to the measurements, samples (0.5–0.7 ml) were thermostated in measurement chamber of the rheometer for 15 min. A temperature 25 ± 0.1°C was maintained using a Thermo Fisher Scientific Accel 500LC thermostat. The measurements were run at 0.1 rpm. Depending on the cone speed and torque the viscosity of the sample was calculated by Reocalc T software. To provide a constant shear stress the rotation was performed for 300 s until the shear rate/shear stress ratio reached an equilibrium.

### Extrusion

The diffusion-limited photofabrication process was based on extrusion of photoinitiator-free bioink into aqueous solution containing free radicals. Bioink was loaded into a sterile syringe with a removable needle that was immersed into 0.066 mM FMN dissolved in PBS. To facilitate a cross-linking process, 33.5-mM TEOHA was added as the co-initiator. The photoinitiator solution was excited by a semiconductor laser at a wavelength of 450 nm and 900 mW power. The excitation uniformity was realized by irradiating the photoinitiator solution, while scanning a 16-mm laser beam across the solution. The external diameter of a fiber was controlled by a nozzle, while the inner diameter was controlled by the non–cross-linked bioink that was adjusted by an exposure dose. Removing non–cross-linked ink was performed in PBS by squashing the fiber from one end.

### Mechanical Tests

The compression test machine EZ-Test EZ-SX (Shimadzu, Japan) equipped with 500-N load cell was employed for mechanical testing of the samples with cylindrical shape. Young’s modulus *E* is directly proportional to the compressive stress to compressive strain ratio:

$$E=Slope\times \frac{L_{0}}{A_{0}},$$ (2)

where *L*_0_ is the gauge length, *A*_0_ is the cross-sectional area of the sample under test, and *Slope* is the ratio of the compressive force to compressive strain evaluated by the method of least squares:

$$Slope=\frac{F}{\Delta L},$$ (3)

where F is compressive force and ΔL is compressive strain.

### Evaluation of Free Radical Generation

The efficiency of the free radical generation by FMN and TEOHA was evaluated using DPPH. To carry out this assay, 0.25-mM solution of DPPH in ethanol was mixed with aqueous solutions of photoinitiators (FMN/TEOHA of ratio ranging from 1/1,000 to 1/127) in equal volumes and placed into a 2-mm quartz cuvette. Then the mixture was exposed to 450-nm laser light (intensity, 450 mW/cm^2^), thoroughly stirred and analyzed using an UV/VIS spectrophotometer Cary 50 (United States) at 200–700 nm range. The reaction of DPPH stable radicals with radicals produced by photoinitiators led to disappearance of DPPH absorption peaks at 340 and 520 nm resulting in color change from deep purple to pale yellow. The time required for DPPH radical depletion defined the efficacy of free radical formation by the FMN/TEOHA photoinitiating complex.

### *In Vitro* Experiments

#### Cell Viability Evaluation

Cell-laden fibers were incubated with Hoechst 33342 and calcein AM (50 µM of each dye in PBS) for 30 min in the CO_2_ incubator. After that, the samples were washed with PBS (pH 7.4) three times and the signal was measured using an inverted fluorescence microscope Motic AE31E (China). More detailed microscopy observations were performed using a fluorescent laser-scanning confocal microscope Zeiss LSM 880 (Jena, Germany). The fluorescence of Hoechst 33342 and calcein AM was analyzed separately using ImageJ software, and cell viability was quantified as the ratio of calcein AM to Hoechst 33342 signals.

#### Hemolysis Study

The hemolysis study was carried out according to Evans et al. (2013). Mice were sacrificed, and 1 ml of blood was collected in a 15-ml conical tube. The blood was centrifuged at 400 g at 20°C for 5 min, and the plasma and the upper layer of leukocytes were gently aspirated by a micropipette. The isolated red blood cells were resuspended in PBS (pH 7.4) and split to Eppendorf tubes (109 cells per each sample). Then the samples were added to the cells for 1 h at 37°C. For positive control, Triton X-100 was added to the cells at the final concentration of 1%. Finally, the tubes were centrifuged (400 g for 5 min), and the absorbance of the supernatants was measured using a plate reader at a wavelength of 540 nm. The absorbance of 1% Triton X-100–treated cells was taken as 100%.

#### Enzymatic Degradation of Hyaluronic Acid Glycidyl Methacrylate

*In vitro* enzymatic degradation of HAGM and cross-linked HAGM hydrogels was studied in the presence of testicular hyaluronidase. The volume of all samples was fixed to 0.5 ml. The concentration of non-modified HA and HAGM in hyaluronidase solutions (64 U/ml) was 4%, and the HAGM hydrogel average mass was 18.5 mg. The enzymatic degradation degree (DD) was evaluated according to Morgan–Elson reaction as described in the study by Asteriou et al. (2001). All samples were incubated in aqueous solution of testicular hyaluronidase at 37°C for 3 h. The enzymatic reaction was terminated by increasing the temperature up to 90°C for 2 min. After cooling the samples to 20°C, Na_2_B_4_O_7_×10H_2_O (0.4 ml, 0.08 M) was added, and the mixture was immediately incubated at 80°C for 3 min followed by cooling to 20°C. Then DMAB (0.5 ml, 50 mg/ml) solution in mixture of 7 v/v parts of glacial acetic acid and 1 volume part of 7 M hydrochloric acid was added and incubated at 37°C for 20 min, with continuous monitoring for the solution coloration. The samples were centrifuged for 3 min at 10,000 g, and absorption spectra at 545 and 585 nm were immediately recorded after the incubation. A calibration curve was built for NAG in the presence of preliminary deactivated hyaluronidase (64 U/ml).

Degradation degree *DD* was calculated according to the expression:

$$DD=\;\frac{C_{m}\left(NAG\right)}{C_{m}\left(sach\right)}\times 100\;\left(\text{\%}\right)\text{,}$$ (4)

where $C_{m}\left(NAG\right)$ is the molar concentration of NAG residues (mM) at HAGM reducing ends that are produced during enzymatic cleavage by hyaluronidase, and $\;C_{m}\left(sach\right)$ is the molar concentration of disaccharides (mM) contained in the tested sample.

## Results and Discussion

### Results

**FIGURE 1:**
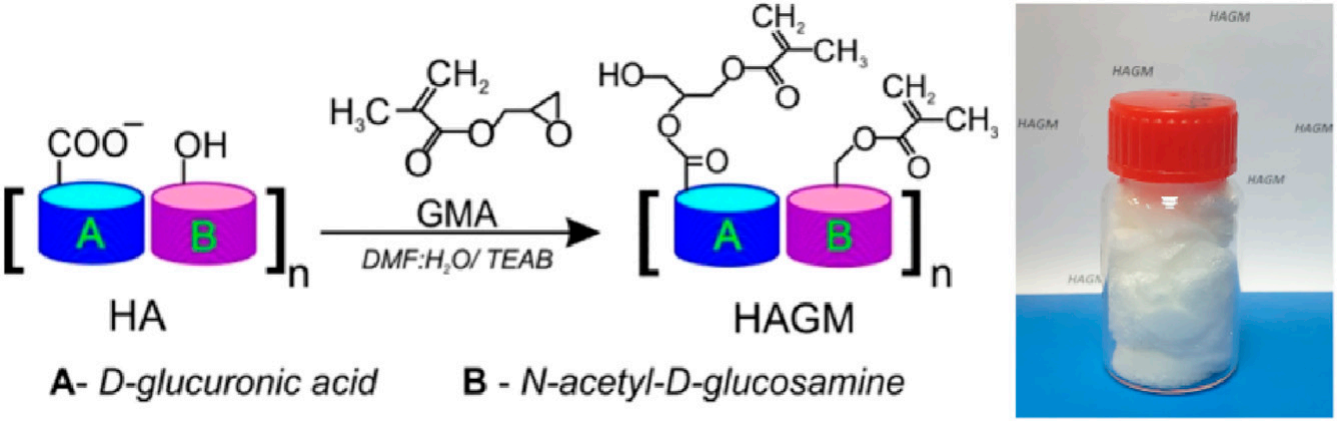
Reaction scheme of HA conjugation with GMA and lyophilized HAGM photopolymer.

Hyaluronic acid was converted to photocurable form by chemical modification in aqueous medium with glycidyl methacrylate to introduce moieties with the vinyl group (Figure 1). The number of vinyl groups is the key parameter affecting cross-linking density of the hydrogel. Therefore, this parameter was precisely controlled in the implemented reaction proceeding as a reversible *trans*-esterification through the primary hydroxyl group and the irreversible epoxide ring-opening conjugation with the carboxylic and hydroxylic groups (van Dijk-Wolthuis et al., 1997; Li et al., 2003; Reis et al., 2009). The reaction parameters, such as the concentration of GMA and reaction time, governed the degree of the modification and enabled HA preparation with percentage of vinyl groups on demand for required cellular responses. We synthesized a number of HAGM modifications with various degrees of substitution (DS) from 11.0 to 69.3%. In order to achieve the lowest DS (11.0%) the reaction was carried out for 1 day with the lowest content of GMA (HA:GMA = 1:5). Increase of the reaction time and/or HA:GMA ratio results in expected magnification of DS up to 69.3% at 5 days of reaction and HA:GMA = 1:35). More detailed dependences of DS on reaction parameters are described in the study by Sochilina et al. (2021).

**FIGURE 2:**
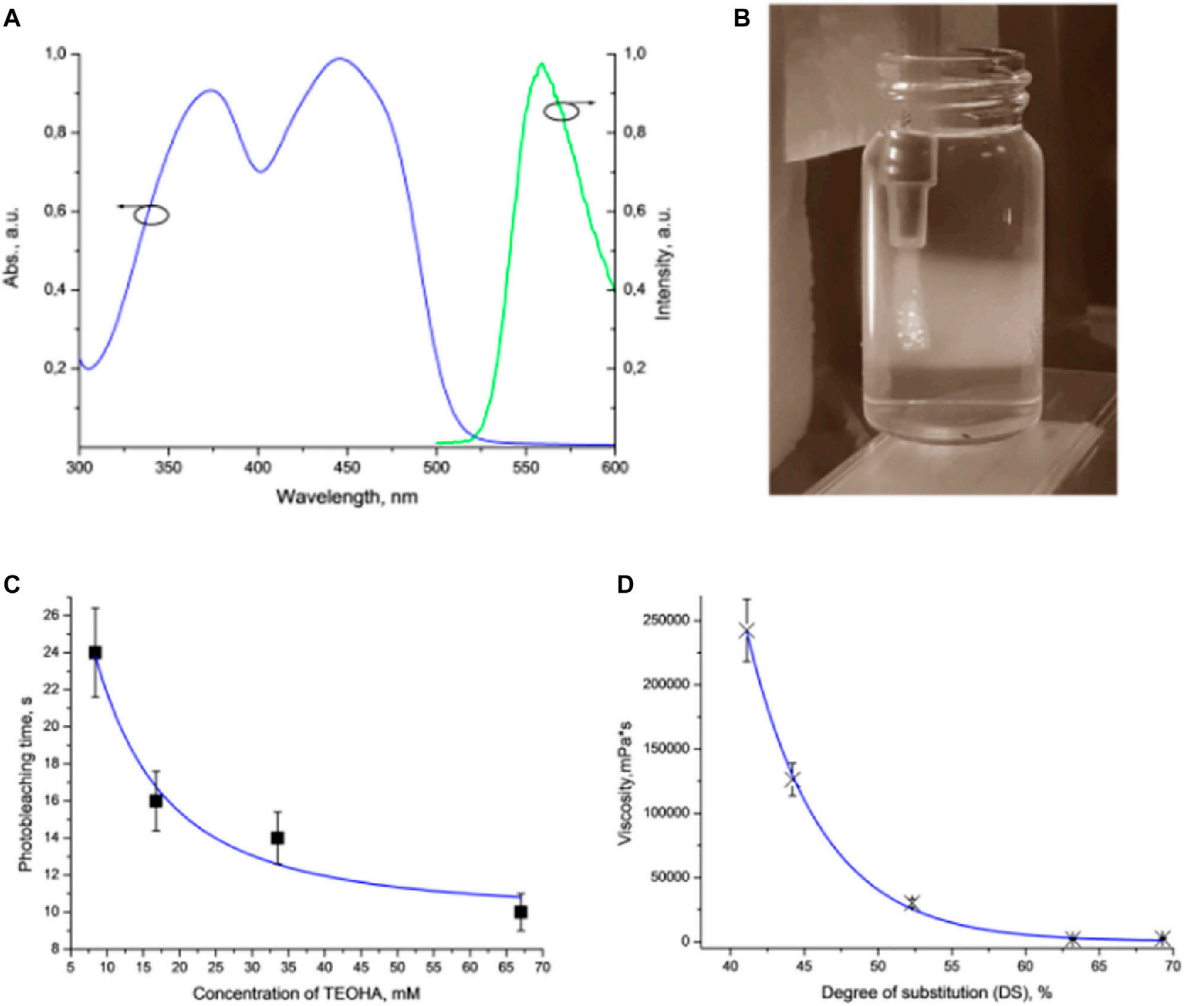
**(A)** Absorption and luminescence spectra of FMN. **(B)** Extrusion of the hydrogel ink into the FMN/TEOHA water solution excited by 450 nm light. Laser excitation visualized as wide illuminated trace. **(C)** Plot of DPPH photobleaching vs. TEOHA concentration in 0.066 mM FMN solution. **(D)** Dynamic viscosity of inks depending on DS of HAGM. Dots indicate experimental data; solid curve represents least squares fitting. All measurements have been performed at least in triplicate, data are presented as mean value, and SD did not exceed 10%.

HAGM aqueous solution was used as the hydrogel ink for extrusion. The photoinitiation was realized by using the FMN/TEOHA complex characterized by an absorption band in the spectral range ∼450 nm (Figures 2A,B). We emphasized the advantage of blue light (>450 nm) as non-toxic for living cells (Opländer et al., 2011), contrary to cytotoxic UV irradiation (Clydesdale et al., 2001) that is necessary for excitation of conventionally employed photoinitiators (BASF, 2021). Implementation of TEOHA as the co-initiator provided homolytic decomposition of vinyl groups, followed by radical cross-linking due to the generation of long-lived radicals. As it is known (Orellana et al., 1999), FMN excitation results in FMN short-lived triplet states (∼1 µs), which can activate a double bond of photopolymer. Amine groups are capable to quench the FMN excited states through an electron transfer from the amine to a flavin molecule, converting them to neutral long-lived (∼100 µs) radical species. These radicals enhance the efficiency of initiation of the vinyl monomer polymerization. Thus, evaluation of an appropriate ratio of FMN/TEOHA is crucial for the photo-cross-linking kinetics of HA containing vinyl bond moieties.

We studied various ratios of FMN/TEOHA in terms of the free radical production using the 2,2-diphenyl-1-picrylhydrazyl (DPPH) radical assay (Magalhães et al., 2008; Alamed et al., 2009). The DPPH radical is a long-lived organic nitrogen radical of deep purple color. DPPH turns yellow, when reducing agents, including radicals, are added. Monitoring the decrease of the DPPH absorbance at 528 nm allowed evaluation of the free radical generation in the mixture of FMN solution with TEOHA at various ratios (Figure 2C). It was found that decreasing the FMN/TEOHA ratio at a constant FMN concentration resulted in a decrease of the photobleaching time. We inferred that an increase of the TEOHA concentration yielded maximum concentration of free radicals, which was capable to promote the effective radical photo-cross-linking. Nevertheless, the FMN/TEOHA ratio lower than 1/300 did not lead to a further rapid decrease of the photobleaching time.

**FIGURE 3:**
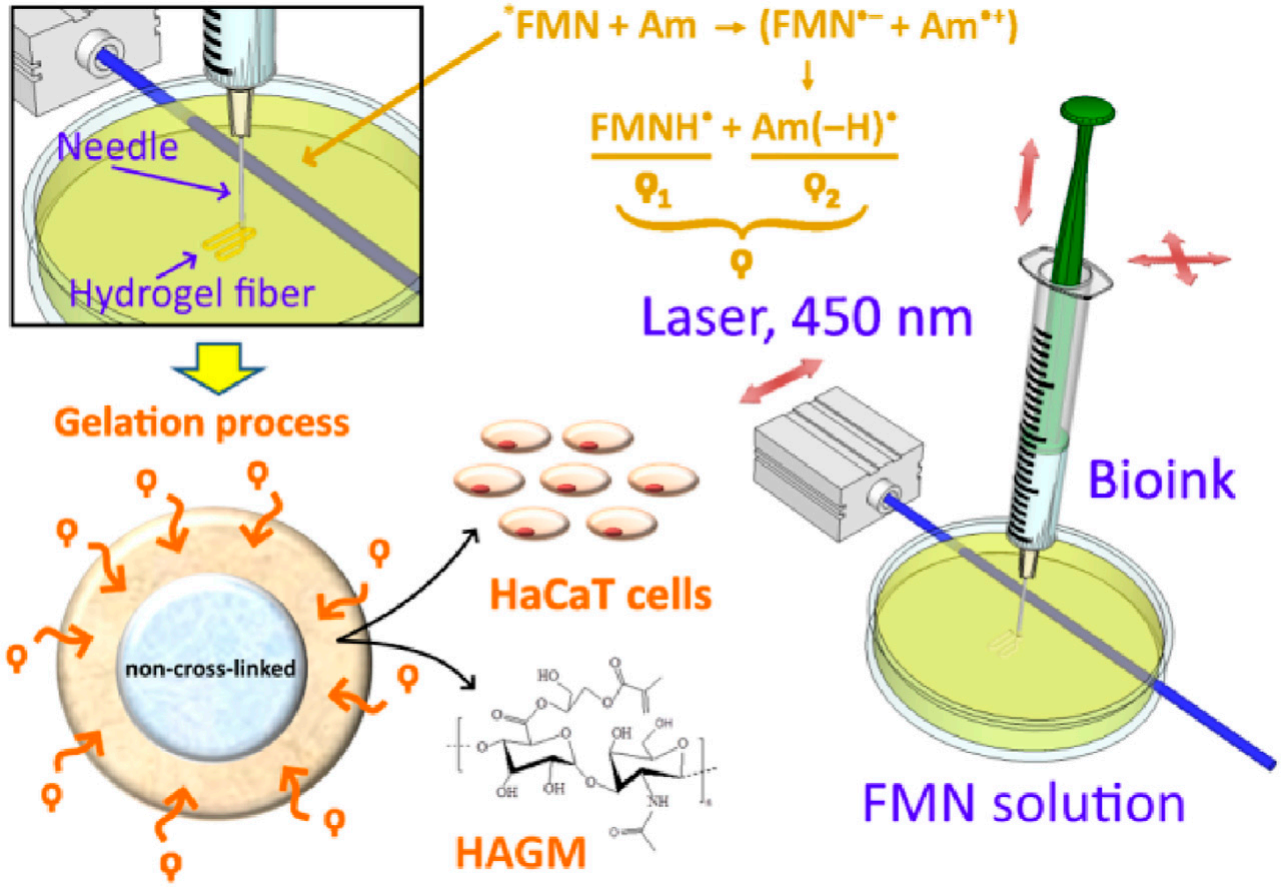
Fabrication process of hollow-core hydrogel fibers by “diffusion-limited photofabrication approach”. *FMN represents the exited triplet state of FMN molecule; Am: amine; (FMN^•–^): semi-reduced flavin; and (Am^•+^): semi-oxidized amine. ϙ represents a long-living species ϙ_1_ and ϙ_2_, where ϙ_1_ is flavin neutral radical (FMNH^•^) and ϙ_2_ is amine neutral radical [Am (–H) ^•^].

The schematic diagram in Figure 3 illustrates key stages of the fiber formation process and radical species formation in the FMN/TEOHA complex under 450-nm laser light irradiation. After photoactivation, radicals permeated through the bioink hydrogel being extruded and triggered cross-linking of the structure from the surface inwards. The penetration depth of the photoexcited radicals determined the extruded fiber cross-linking cross-section profile. Unlinked ink in the central part of structure was removed by dissolving in water. As a result hollow-core structures were produced.

It should be noted that the viscosity of extruded ink is the key parameter limiting the fabrication process. Permeation of the photoexcited radicals in the depth of the extruded volume followed by hydrogel cross-linking must occur prior to complete dissolution of the bioink in the aqueous solution of the FMN/TEOHA complex. To satisfy this condition, the viscosity of the bioink must be higher than 40,000 mPa•s and can be controlled by adjusting the concentration of the polymers, changing HAGM molecular weight or employing HAGM with variable DS (Figure 2D). The degree of substitution (DS) in HAGM used in inks (HAGM 20 wt%, PEG-DA 5 wt%) rising up to 60% led to a viscosity decrease, which is likely to associate with the changes in swelling caused by implementation of hydrophobic moieties in the polymer chain. As a result, the more hydrophobic polymer adsorbs less water, leading to mobility of polymer chains and the shear stress decrease, which translates into a viscosity decrease. Further DS increase probably does not alter the polymer hydrophobicity and, consequently, the viscosity. Therefore, we prepared bioinks containing HAGM (molecular weight ∼100 kDa, DS 46%) 20 wt% and PEG-DA (molecular weight 575 Da) 5 wt%, which resulted in the dynamic viscosity of ∼90,000 mPa•s. This composition ensured uniform extrusion of bioinks and controllable cross-linking in diffusion-limited photofabrication process.

**FIGURE 4:**
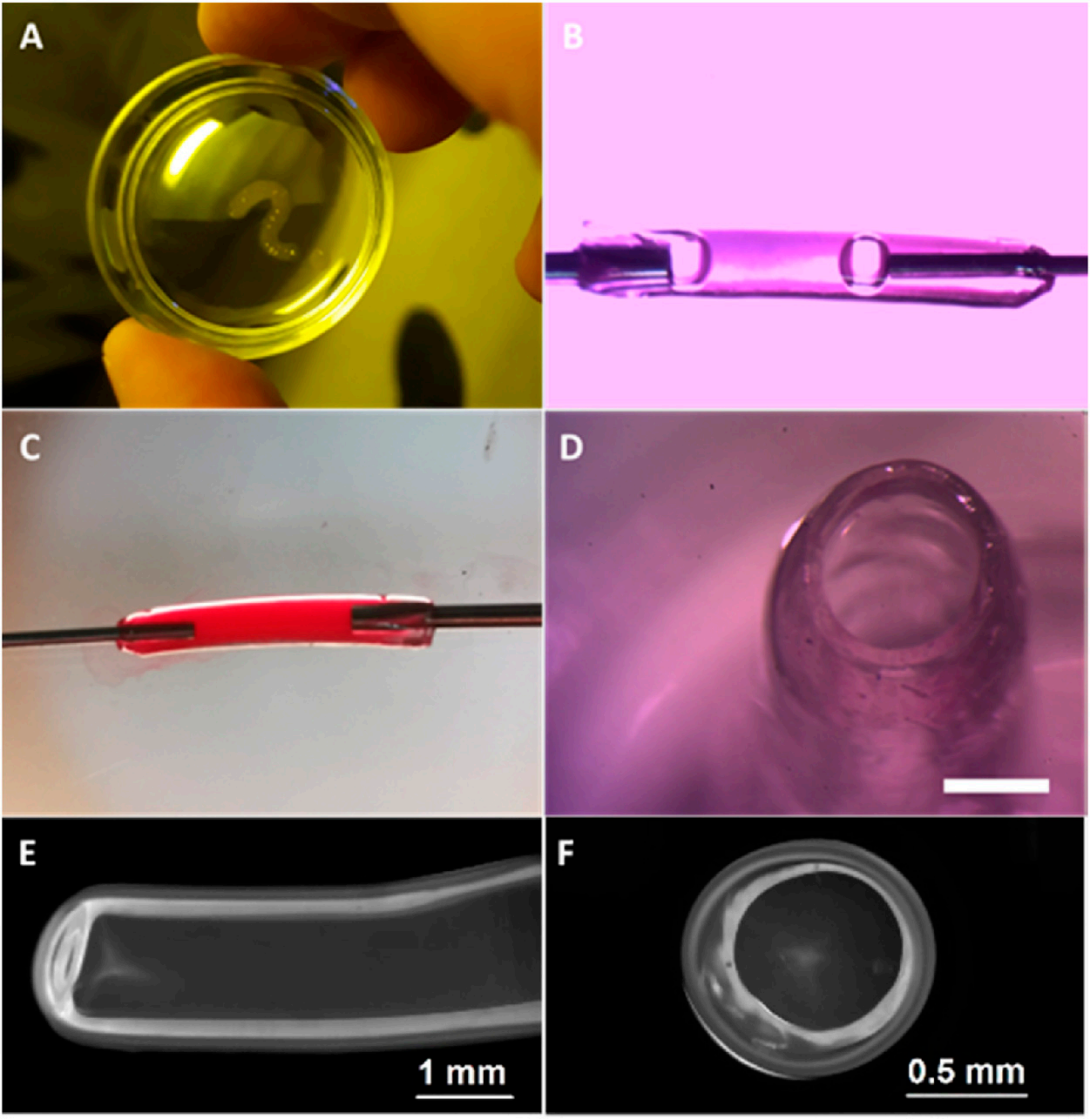
Image of fabricated hydrogel structures. **(A)** Structure printed with the shape of “question mark sign”. **(B)** Bubbled fluid flow through the fiber and **(C)** colorized liquid flux flowing through the hollow-core fiber. **(D)** Cross-sectional view of the fiber after developing, scale bar 1 mm. **(E)** Fiber produced by 600 µm nozzle and **(F)** its cross-sectional view.

Figure 4 illustrates images of hydrogel structures extruded in aqueous solution of 0.066-mM FMN and 33.5-mM TEOHA under excitation with light at 450 nm. Fibers were extruded through 1.3 mm nozzle at a rate of 0.15 ml/min. Aiming to form a hollow-core fiber, the samples after 3.5 min exposition were placed in PBS for developing to allow uncross-linked hydrogel clear from the fiber core volume by dissolution. As a result, a hollow-core fiber was formed with 3-mm outer diameter and 350-µm wall thickness. Figure 4A illustrates structure with the shape of “question mark sign” printed on the substrate. In order to demonstrate possibilities of retention and motion of the fluid the hollow-core fiber was connected to nozzles. Fiber ends were treated with pressurized air to partially dry and tightening of the hydrogel to fit to the nozzles. Figures 4B,C demonstrate the colorized liquid flux flowing through the fiber with and without bubbles. End facet view of the fiber is presented in Figure 4D. To estimate the cross-linking rate, we formed the fibers with extrusion velocity of 0.15 ml/min through a wide 2.4 mm nozzle. It was found that an increase of the cross-linking process time from 3 to 4.5 min increased the average fiber wall thickness from 330 to 380 µm. Increasing the cross-linking time up to 15 min resulted in solid-core fiber formation. The minimal wall thickness of hollow-core fiber was obtained at extrusion through the 600 µm nozzle. Figures 4E,F illustrate the cross-section of a hollow-core fiber with wall thickness ∼300 μm. Note: HAGM is the material that is subjected to swelling after formation. The extrusion and subsequent cross-linking occur in the water medium, so we avoided the negative factors associated with swelling of the final structure, and water loss by samples is excluded in our approach.

**FIGURE 5:**
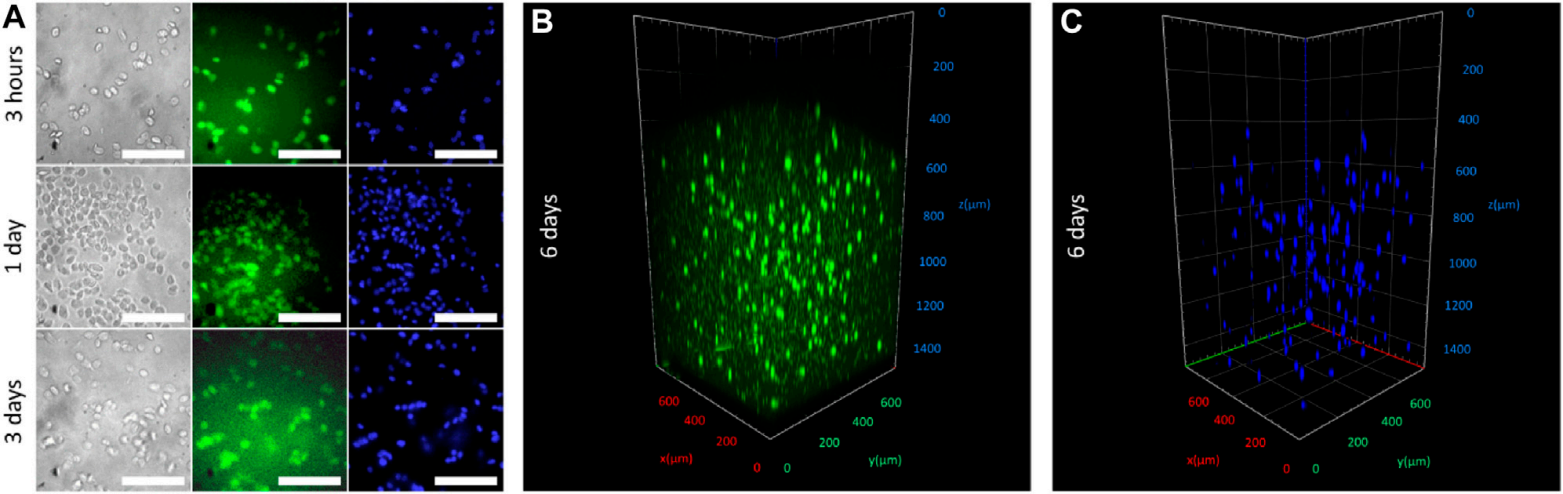
**(A)** *In vitro* imaging after 3, 24, and 72 h incubation of HaCaT-laden fibers stained with calcein AM (fluorescence labeled alive cells, in green) and Hoechst 33342 (cell nucleuses, in blue). Scale bar 100 µm. **(B,C)** Fluorescence confocal imaging of fibers acquired on day 6 of incubation stained with calcein AM and Hoechst 33342.

To demonstrate the cell-friendly gelation process, we loaded the bioink with HaCaT cells, immortalized human keratinocytes, which are often used in bioprinting due to their high viability and survivability (Koch et al., 2012; Jeffries et al., 2020; Boonyagul et al., 2021). Free radicals penetrating from the solution to the interior of a bioink hydrogel structure were spent for the activation of the HAGM double bonds. Although using FMN as a photosensitizer for cell damage is well known (Baier et al., 2006; Akasov et al., 2019), we speculate that the exhaustion of potentially cytotoxic free radicals in the hydrogel volume allowed cells embedded in the hydrogel structure volume to escape negative influence. In other words, reactive oxygen species (ROS) generated at the photoactivation FMN/TEOHA complex cannot cause cells oxidative stress because the free radicals quenching took place predominantly by HAGM molecules. Figure 5 represents microscopy images of cell-laden fibers extruded through 2.4-mm nozzle on 3 h and 1, 3, and 6 days of structure incubation. Cell-permeant dyes calcein AM and Hoechst 33342 were used to determine cell viability and nuclei staining, respectively. Fluorescence imaging indicated the cell viability for 6 days and confirmed cell-amiable hydrogel gelation. Since fluorescence of calcein AM could be used to quantify cell viability, we normalized the calcein AM fluorescent signal to Hoechst 33342 one. We found that calcein AM mean fluorescence significantly increased from 34.5 ± 4.7 on day 3–43.1 ± 7.1 (*p* < 0.05), while the calcein AM/Hoechst 33342 ratio increased from 1.09 on day 3 to 1.35 on day 6 (Supplementary Section S1). This observation could be explained with the increase of viability in cell population. It should be noted that the total cell amount calculated using Hoechst 33342 fluorescence increased insignificantly (from 31.8 ± 5.0 on day 3–32.0 ± 2.3 on day 6) and this could be explained with the slowdown in cell growth in the dense hydrogel microenvironment. We found that 95–100% of cells stained with Hoechst 33342 had also been labeled with calcein AM that confirmed a high survival rate of cells during extrusion, photopolymerization, and followed incubation. This survival rate did not concede the viability of cells in previously published researches devoted to 3D printing of cell-laden scaffolds (Sharma et al., 2020; Hull et al., 2021).

Additionally, hollow-core structures were tested on hemocompatibility. No toxicity was found for as-produced hydrogel structures containing HAGM 20 wt% and PEG-DA 5 wt% (see Supplementary Section S2). Since the concentration of PEG-DA in the bioink appeared to be an instrumental to control the mechanical properties of the produced structures (adding PEG-DA in HAGM:PEG-DA concentration as 2:1 leads to 3-fold increase of hydrogel Young’s modulus) (Savelyev et al., 2017), the hydrogel with up to 15 wt% concentration of PEG-DA was also studied and no detectable toxicity changes were revealed.

Biodegradation is an important requirement for hydrogel samples to control the longevity of an artificial implant *in vivo*. To evaluate the biodegradation property of as-produced extruded bioink hydrogels, we compared the stability of HAGM before and after the photo-cross-linking *in vitro* using bovine testicular hyaluronidase, which is known to cleave HA within the living organism (see Supplementary Section S3). Morgan–Elson colorimetric reaction experiments with HAGM 4% solution revealed a decrease of DD from 56 to 27% which corresponded to an increase of the GMA substitution in HAGM with DS from 0 to 63%. We hypothesized that the observed biodegradation was caused by formation of steric hindrance for enzymes during the substrate hydrolysis. A similar pattern of the DD change of the HAGM hydrogels depending on DS was confirmed. In the case of hydrogels, an increase in the degree of the cross-linking can lead to the formation of even greater steric hindrance for the enzyme. The hindered enzymatic degradation of HAGM hydrogels is associated with the prolonged lifetime of such hydrogels in the living body. Thus, variation of DS in initial HAGM appeared to be a promising parameter to control our hydrogel structure lifetime *in vivo*.

We performed measurements of the Young’s modulus of hydrogel samples. It should be noted, that drying of the samples was excluded during the measurements. The hydrogel containing HAGM 20 wt% and PEG-DA 5 wt% indicated Young’s modulus ∼1.6 ± 0.4 MPa. This value of Young’s modulus falls in the range of Young’s moduli of soft tissues under physiologic conditions including blood vessels (0.701–10.7 MPa) (Gao et al., 2007; Faturechi et al., 2019).

### Discussion

Poor processability and uncontrollable mechanical properties of native polymers limit their application scope in tissue engineering. A number of synthetic polymers, such as polylactic acid (Savioli Lopes et al., 2012), poly(lactic-co-glycolic acid (Castillo-Dalí et al., 2015), and modified polyvinyl alcohol (Velutheril Thomas and Nair, 2019) have been proposed to realize tunable mechanical and biodegradability properties. However, poor biocompatibility of these synthetic materials remains their main drawback. Hybrid composite material that comprises native and synthetic polymers has potential to overcome the limitations inherent to natural polymer while retaining the advantages of synthetic ones. Natural polymers (hydrogels) are derived from alginate, collagen, gelatin, chitosan, agarose, and hyaluronic acid. Among these, we chose endogenous hyaluronic acid due to its properties such as hydrophilicity and biocompatibility. The polymer can be modified with various moieties allowing for a multitude of cross-linking chemistries (Day et al., 2018), for example, glycidyl methacrylate.

Implementation of HAGM as the key component of the photocurable composition improved its toxicity and processability features. For instance, high viscosity of the bioink on the base of HAGM (dynamic viscosity >90,000 mPa•s) improved its printability (Chimene et al., 2020). Moreover, using HAGM in a combination with PEG-DA opens a possibility to produce hydrogel with controllable mechanical properties using the cell-friendly cross-linking. Taking into account our previous study of hydrogels on the base of methacrylated hyaluronic acid (Savelyev et al., 2017), we believe that stiffness of the hydrogel can increase >10-fold by simply changing the concentration of macromolecules. It either can be adjusted by changing the molar weight of the initial polymers or by varying DS of HA functional groups by GMA.

It should be noted that Xu et al. (2020) have proposed and demonstrated an extrusion method for fabrication of biomimetic vessels using conjugated polymers. Formation of small diameter and heterogeneous bilayer blood vessel-like constructs was carried out by using gelatin methacryloyl and the photoinitiator Irgacure 2959 by employing a layer-by-layer photo-cross-linking process. Every four layers produced by an integrated tissue–organ printing (ITOP) system were cross-linked by using UV (365-nm) radiation. We believe that the reported layer-by-layer photo-cross-linking using UV-cured Irgacure 2959 limits an application scope of this approach by exerting an excessive stress on cells. Coaxial technology represents another method of hollow-core tube fabrication. This method suffers from the requirement of machining coaxial nozzles specific for each hollow-core fiber configuration and makes control over the thickness of tube walls difficult. Our approach based on direct single-step extrusion of a hydrogel structure into aqueous solutions containing free radicals was demonstrated to produce controllable cell-amiable hollow-core tubular structures. Moreover, the presented approach has a potential to be implemented in a combination with coaxial nozzle due to advantages in combining several cell types in a highly ordered pattern making the fiber fabrication more versatile and demanded.

In our study, the minimal and maximal diameters of hollow-core fibers were limited by the diameters of nozzles, while the width was adjusted by exposure time. We assumed that the maximum diameter of fiber is unlimited, while the minimal diameter of fiber is affected by the technical limitations. Extrusion of fibers through nozzles 1 mm in diameter is challenging because the fiber starts curling, especially when small gas bubbles remain in the depth of bioinks, so the additional degassing procedure is required. Therefore, it becomes difficult to produce uniform samples. The minimal wall thickness should be chosen enough to provide adequate mechanics of the tube in order to avoid its collapse. The maximal wall thickness is limited by the exposure time. In the experiment for the solid-core fiber with diameter ∼3 mm the exposure time exceeded 15 min. In other words, for translating our approach to 3D printing technology, we speculated that it is maximally acceptable time for photo-cross-linking of the structure. Nevertheless, we believe that our technology is well applicable for fabrication of fibers in a wide range of outer/inner diameters suitable for mimicking blood vessels or bile ducts. Combination of the extrusion process with automatic removal of the fiber from the solution containing free radicals to a developing tank will enable fabrication of samples with virtually unlimited length. Furthermore, changing the time of the cross-linking process in the solution containing free radicals enabled accurate control over the inner diameter ranged from 0% (solid core) to 88% of the outer diameter. As-produced hydrogel hollow-core fibers are easily integrated in more complicated structures or used as a part of tissue engineering construction to facilitate the vascularization process. We believe our method represents a versatile fabrication platform for diverse tissue engineering applications. Thus, our cell-laden bioink diffusion-limited photofabrication is superior in comparison with the existing tissue engineering approaches, where the scaffold printing is followed by cell seeding, which made the process time-consuming. Our technique is facile, single-step, and more affordable.

However, an uptake of our technology in the life sciences and clinical applications hinges on several improvements. For example, permeability and porosity control of the hydrogel warrants further studies in the future. The problem of interconnection of native and artificial vessels is an inherent problem of biomimetic vessels, including our reported technology. Moreover, the biodegradation of grafts based on hollow-core fibers must be experimentally estimated in the full context of a living organism. Adaptation of diffusion-limited photofabrication process for implementation in 3D printing is also required (Supplementary Section S4).

## Conclusion

In this study, we reported a versatile facile scalable technique for rapid bioink prototyping of polymer fibers. A derivatized hyaluronic acid (HAGM) hydrogel structure was extruded into a solution of photoinitiators based on flavin mononucleotide and was irradiated with cell-amiable blue light to generate free radicals required for the hydrogel cross-linking. The photo-cross-linking started at the curved surface of the structure being extruded and yielded hollow-core hydrogel fibers of controllable inner and outer diameters. Phototoxic stress to cells embedded into the bioink hydrogel structure was minimized due to scavenging of free radicals during the cross-linking process and allowed demonstration of biopolymer tissue constructs. As such, cross-linked HAGM hydrogel fibers present favorable microenvironment for living cells, while the fabrication technology in comparison to standard hydrogel extrusion enhances cell viability during photopolymerization. A hemolysis study confirmed a potential of hydrogel hollow-core fibers to be used for treatment of vascular disorders and reconstructive surgery. Our demonstrated single-step hollow-core fiber production technology has potential to overcome long-standing bottleneck problem of vessel engineering to advance 3D printing in the future.

## Data Availability

The original contributions presented in the study are included in the article/Supplementary Material; further inquiries can be directed to the corresponding authors.
