# Supplementary material for: Facile Cell-Friendly Hollow-Core Fiber Diffusion-Limited Photofabrication
Source: Front Bioeng Biotechnol. 2021 Dec 3;9:783834. doi: 10.3389/fbioe.2021.783834 (PMC8678487; doi:10.3389/fbioe.2021.783834)
Supplement: Supplementary file 2 [file Image1.pdf]

Supplementary Material

Section 1

Study of hemolytic toxicity for as-prepared hydrogel structures is presented in Supplementary Fig. 1.

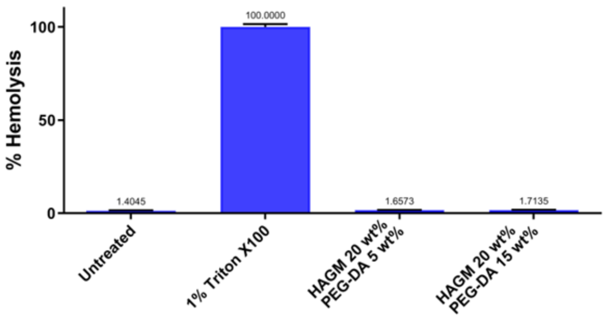

Supplementary Figure 1. Hemolysis assay of hydrogel samples, containing 20 wt% of HAGM and 5-15 wt% of PEG-DA after 1-h incubation. Data are the mean ± SD of 3 measurements. Untreated cells were used as negative control. Triton X-100 (1 %) was used as positive control, the absorbance of 1% Triton X-100 treated cells was taken as 100 %.

Section 2

To study biodestruction of the HAGM *in vitro,* we have used bovine testicular hyaluronidase. Since hyaluronidase cleaves 1-4 bonds in HA, leaving NAG degradation products at the reducing end, the degree of degradation can be measured as the percentage of NAG at the reducing end to the total number of disaccharide units. To calculate NAG at the reducing end, the Morgan-Elson colorimetric reaction has been used, in which the Ehrlich reagent (DMAB) forms a cherry-colored compound with the so-called chromogen III (transformation product of N-acetylaminosugars) (Morgan and Elson, 1934). This reaction is highly specific since the appearance of intense characteristic peaks at 545 and 585 nm is possible only with N-acetylaminosugars.

The study of the enzymatic degradation of modified HA and hydrogels based on it has been carried out regarding to DS of HAGM, which is considered as the percentage of attached GMA groups (determined by the colorimetric method with potassium permanganate per 100 disaccharide units). The HAGM samples with different DS (from 11 % to 63 %) have been prepared to study the enzymatic degradation. The significant decrease of DD has been indicated as a result of the GMA content increase in HAGM composition (see Supplementary Fig. 2). This could be explained due to appearance of steric difficulties for the enzyme during the substrate hydrolysis.

The character of DD change of the HAGM hydrogels depending on DS is presented in Supplementary Fig. 3. Although the general tendency of biodegradation of HAGM solution and its composition in the hydrogel is the same, they cannot be compared. In the HAGM solution the

reaction takes place in a homogeneous medium, while in the case of the hydrogels, mostly from the surface of the hydrogel, which can change the activity of the enzyme.

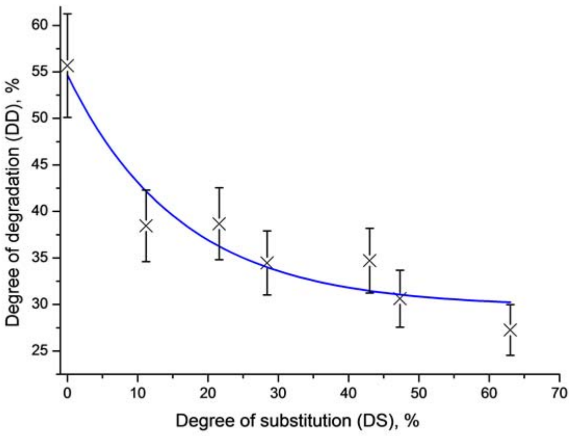

Supplementary Figure 2. DD of HAGM 4 % solutions depending on DS. Dots indicate experimental data, solid curve represents least squares fitting. All measurements have been performed at least in triplicate, data are presented as mean value, SD did not exceed 10 %.

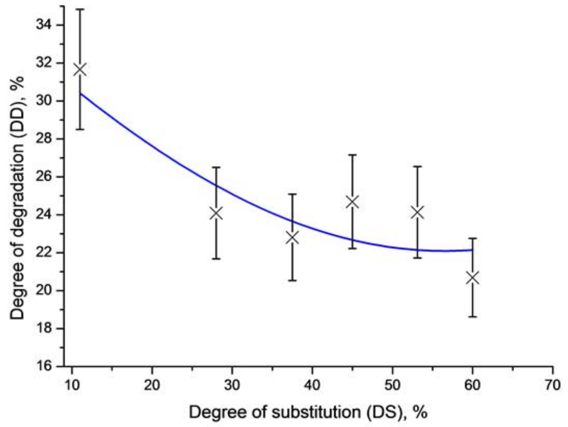

Supplementary Figure 3. DD of HAGM hydrogels depending on DS. Dots indicate experimental data, solid curve represents least squares fitting. All measurements have been performed at least in triplicate, data are presented as mean value, SD did not exceed 10 %.

Section 3

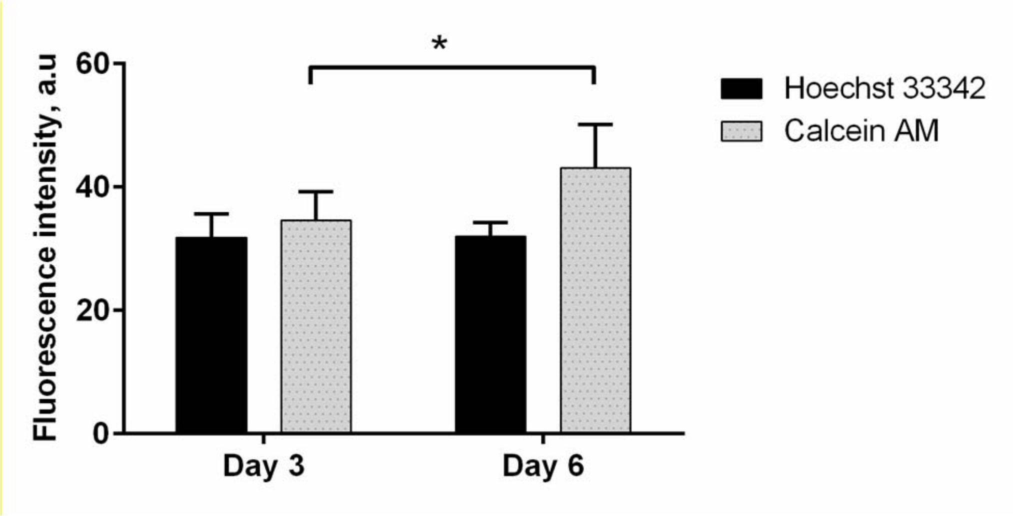

Supplementary Figure 4. The fluorescent signal of Hoechst 33342 and Calcein AM obtained from a panel (n > 10) cell-laden scaffold images on day 3 and day 5 of incubation. The images were analyzed using Image J software; * $p<0.05$ in Mann-Whitney U test.

Section 4

Taking into account the prospects of diffusion limited photofabrication for 3D printing we have created our own printer. Printing nozzle has a possibility of movement in 3 axes. Printing head was adopted for fabrication of 3D hydrogel structures using HAGM bioinks. (see Supplementary Figure 5).

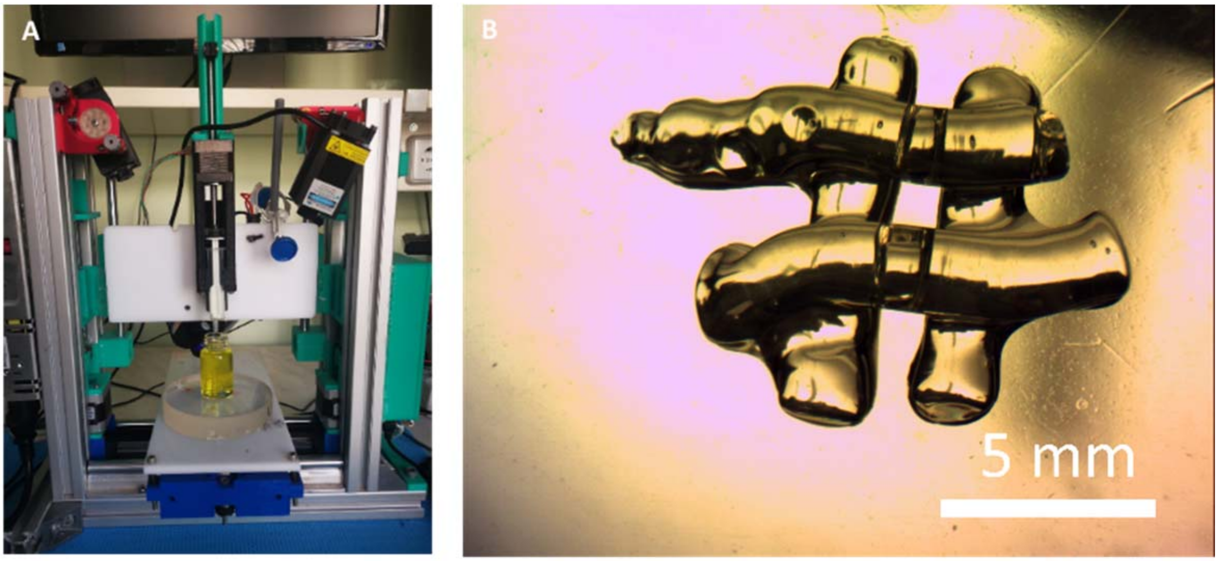

Supplementary Figure 5. Owned-designed 3D-printer based on inject module equipped with 450 nm laser and motorized coordinate table (A) and produced hyaluronic hollow tube 3D structure (B).

References

Morgan, W. T. J., and Elson, L. A. (1934). A colorimetric method for the determination of N-acetylglucosamine and N-acetylchrondrosamine. *Biochem. J.* 28, 988–995. doi:10.1042/bj0280988.
